# A New Murine Model of Primary Autoimmune Hemolytic Anemia (AIHA)

**DOI:** 10.3389/fimmu.2021.752330

**Published:** 2021-11-15

**Authors:** Flavia Dei Zotti, Annie Qiu, Francesca La Carpia, Chiara Moriconi, Krystalyn E. Hudson

**Affiliations:** Columbia University Irving Medical Center, Department of Pathology and Cell Biology, New York, NY, United States

**Keywords:** red blood cell, erythrocyte, autoimmune hemolytic anemia, tolerance, autoimmune disease, primary autoimmune hemolytic anemia, RBC (red-blood-cell), idiopathic autoimmune hemolytic anemia

## Abstract

Loss of humoral tolerance to red blood cells (RBCs) can lead to autoimmune hemolytic anemia (AIHA), a severe, and sometimes fatal disease. Patients with AIHA present with pallor, fatigue, decreased hematocrit, and splenomegaly. While secondary AIHA is associated with lymphoproliferative disorders, infections, and more recently, as an adverse event secondary to cancer immunotherapy, the etiology of primary AIHA is unknown. Several therapeutic strategies are available; however, there are currently no licensed treatments for AIHA and few therapeutics offer treatment-free durable remission. Moreover, supportive care with RBC transfusions can be challenging as most autoantibodies are directed against ubiquitous RBC antigens; thus, virtually all RBC donor units are incompatible. Given the severity of AIHA and the lack of treatment options, understanding the cellular and molecular mechanisms that facilitate the breakdown in tolerance would provide insight into new therapeutics. Herein, we report a new murine model of primary AIHA that reflects the biology observed in patients with primary AIHA. Production of anti-erythrocyte autoantibodies correlated with sex and age, and led to RBC antigen modulation, complement fixation, and anemia, as determined by decreased hematocrit and hemoglobin values and increased reticulocytes in peripheral blood. Moreover, autoantibody-producing animals developed splenomegaly, with altered splenic architecture characterized by expanded white pulp areas and nearly diminished red pulp areas. Additional analysis suggested that compensatory extramedullary erythropoiesis occurred as there were increased frequencies of RBC progenitors detectable in the spleen. No significant correlations between AIHA onset and inflammatory status or microbiome were observed. To our knowledge, this is the first report of a murine model that replicates observations made in humans with idiopathic AIHA. Thus, this is a tractable murine model of AIHA that can serve as a platform to identify key cellular and molecular pathways that are compromised, thereby leading to autoantibody formation, as well as testing new therapeutics and management strategies.

## Introduction

Autoimmune hemolytic anemia (AIHA) is a disorder characterized by destruction of red blood cells (RBCs) by pathogenic anti-erythrocyte autoantibodies. Hemolysis leads to significant reductions in RBC numbers, leading to inadequate tissue oxygenation and patients present with pallor, fatigue, hemoglobinuria, anemia and splenomegaly (1, 2). Similar to other autoimmune diseases, AIHA is more prevalent in women (3). Treatment strategies (e.g., immunosuppressants, splenectomy, etc.) have variable success and relapse rates can be as high as 50%, with mortality in 10% of cases (4–6). Moreover, supportive care through RBC transfusions is challenging as most autoantibodies recognize ubiquitous RBC antigens, thereby making virtually all donor RBC units crossmatch incompatible and risking further exacerbation of the immune responses to RBCs (7, 8).

The etiology of AIHA is unknown. And, although some cases are secondary and are associated with lymphoproliferative disorders, infections, and cancer immunotherapy, >50% of AIHA cases are idiopathic (9, 10). As RBCs carry out essential functions required for life, it would be predicted that tolerance to RBC autoantigens would be stringent. Unexpectedly, loss of tolerance to RBCs occurs frequently; 0.1% of healthy blood donors have detectable non-hemolytic autoantibodies bound to their RBCs and this increases up to 8% in hospitalized patients (11, 12). Elucidation of the initiating events that lead to development of AIHA have relied on both observations in human AIHA patients and murine models. With these studies, breakdown of tolerance to RBC autoantigens has been shown to be multifactorial (e.g., genetics as observed in NZB mice, immune dysregulation as demonstrated in the Playfair and Marshall-Clarke model, etc.) (13, 14). However, despite several AIHA murine models, one barrier to elucidating the initiating events leading to the failure of RBC tolerance is the lack of a model that accurately reflects primary AIHA.

Our group and others have used an RBC transgenic mouse to study immune responses to erythrocyte antigens: the HOD mouse model. HOD mice express an RBC-specific triple fusion protein consisting of hen egg lysozyme (HEL), ovalbumin (OVA) and human blood group molecule Duffy (HEL-OVA-Duffy; HOD) (15); HEL and OVA are well-characterized B and T cell antigens. The HOD antigen is expressed under an RBC-specific promoter and is detectable only on RBCs, not on leukocytes or platelets, and at a similar level to naturally occurring RBC antigens (15). Tolerance to the HOD RBC autoantigen is robust despite the presence of detectable HOD-reactive B and T cells. HOD mice do not make HOD-specific autoantibodies upon immunization with either HEL or OVA self-proteins in complete Freund’s adjuvant (CFA), a regimen that readily elicits high titer antibodies in wild-type B6 animals and can induce autoimmunity in some settings on its own (16–18). Additional studies revealed that while HOD autoreactive CD4+ T cells are detectable in HOD mice, they are non-functional (16). Importantly, traditional CD4+FoxP3+CD25+ regulatory T cells are not required for maintenance of T cell tolerance to RBCs in HOD mice (19). In contrast to the observed stringent T cell tolerance in HOD animals, B cell tolerance is incomplete. The HOD antigen, specifically the N-terminal portion of OVA, contains OVA_323-339_, an epitope recognized by CD4+ T cells from T cell receptor (TCR) transgenic OTII mice (20); and, adoptive transfer of CD4+ OTII T cells, thereby circumventing T cell tolerance, into HOD animals stimulates autoreactive B cells to secrete autoantibodies against HOD RBCs (16). Thus, in HOD mice, B cell tolerance is incomplete and T cell tolerance is the stopgap to RBC autoimmunity. The described studies characterized endogenous autoreactive lymphocytes, which were limited due to low precursor frequencies. As such, to gain additional mechanistic insight into the establishment and breakdown of tolerance to RBC autoantigens, HOD mice were bred with OTII TCR transgenic animals (HODxOTII F1 mice consisting of autoreactive genotype HOD+OTII+ mice and HOD-OTII+ littermate controls). Initial analysis of HOD+OTII+ animals revealed that autoreactive CD4+ T cells are non-functional and are subject to peripheral tolerance (e.g., deletion, anergy, and induced regulatory T cells), and upregulate markers associated with anergy and exhaustion (21). However, unexpectedly, a subset of HOD+OTII+ animals spontaneously generated HOD autoantibodies upon aging. Herein, we describe a new murine model of AIHA, which have similar presentation as human patients with primary AIHA, including decreased hematocrit, splenomegaly, extramedullary hematopoiesis, and female predominance. This model will be fruitful in identifying which T cell tolerance pathways are required to prevent autoimmunity and will also serve as a platform to test new therapeutics.

## Materials and Methods

### Mice

C57BL/6 (B6) (C57/BL6NCr; stock #556) mice were purchased from NCI Charles River. OTII (B6.Cg-Tg (TcraTcrb)425CBn/J; stock #004194) mice were purchased from the Jackson Laboratory. HOD mice were developed and described previously (15). HOD and OTII mice were bred to generate F1 animals, consisting of HOD+OTII+ and HOD-OTII+ littermate controls. Mice were phenotyped by flow cytometry. OTII.Rag2^−/−^ mice (B6.129S6-Rag2tm1FwaTg (TcraTcrb) 425Cbn, stock #1896) were purchased from Taconic Biosciences. TCR75 mice were a gift from Dr. Bucy (University of Alabama Birmingham) (22). Mice were maintained on standard rodent chow and water in a light- and temperature-controlled, pathogen-free environment. Protocols were approved by the BloodworksNW and Columbia University Institutional Animal Care and Use Committees (IACUC).

### Flow Crossmatch

Blood was drawn by retro-orbital puncture from experimental mice. Collected sera were diluted 1:100 and incubated with HOD or B6 RBCs (1:100) in fluorescence activated cell sorter (FACS) buffer (phosphate-buffered saline + 0.2 mg/mL bovine serum albumin [Sigma-Aldrich] + 0.9 mg/mL ethylenediaminetetraacetic acid [Sigma-Aldrich]) for 30 minutes at 4°C. Samples were washed with FACS buffer 3 times followed by incubation with a secondary antibody (diluted 1:100 in FACS buffer) for 30 minutes at 4°C. Secondary reagents included goat anti-mouse Ig APC-conjugated (Southern Biotech), goat anti-mouse IgM APC-conjugated, IgG Fc*γ* subclass 1 R-Phycoerythrin-conjugated, IgG Fc*γ* subclass 2b FITC-conjugated, IgG Fc*γ* subclass 2c APC-conjugated and IgG Fc*γ* subclass 3 Brilliant Violet 421-conjugated (Jackson ImmunoResearch). Samples were washed with FACS buffer 3 times and then acquired using an Attune NxT flow cytometer and analyzed with FlowJo software.

### Peripheral RBC Analysis

Whole blood was collected by retro-orbital puncture into heparinized glass capillary tubes. To detect HOD antigen expression, RBCs were incubated with purified MIMA-29 followed by anti-mouse Igs APC, as previously described (19). To assess C3 complement deposition, RBCs were incubated with anti-C3 biotin antibody followed by streptavidin-APC secondary reagent. Detection of antibodies bound to RBCs (DAT) was determined by staining with goat anti-mouse Ig APC. To determine hematocrit, whole blood samples were centrifugated in a microhematocrit centrifuge. Then, the ratio of the volume occupied by packed RBCs to the volume of whole blood was measured and expressed as a percentage. To determine hemoglobin concentration, whole blood samples were incubated with Drabkin’s reagent (Sigma-Alridch) for 10 minutes, centrifuged and absorbance at 540nm was recorded. The total hemoglobin concentration (gr/dL) was determined using a calibration curve. To count reticulocytes, peripheral blood was resuspended in FACS buffer and stained with antibodies against Ter119 and CD71. The percentage of Ter119+CD71^high^ reticulocytes was calculated from total Ter119+ RBCs.

### Cytokines

Sera samples from experimental mice were diluted 20-fold. The cytokines IFN-*γ*, CXCL1, TNF-α, CCL2, IL-12, CCL5, IL-1β, CXCL10, IL-10, IL-6, IFN-β, IFN-α and GM-CSF were quantified utilizing fluorescence-encoded beads based multiplex assay (Legendplex™ Multi-analyte flow assay kit; BioLegend). Samples were acquired using Attune flow cytometer and analyzed with FlowJo software.

### Spleen Pathology

Spleens were collected and weighed. In some experiments, spleens were fixed in formalin, sectioned, and stained with hematoxylin and eosin (H&E). Slides were imaged with an Olympus microscope and images were processed with Bioquant Osteo 2012 software. All captured images are shown with their respective magnification.

### RBC Development and Hematopoietic Precursor Detection in Spleen, Bone Marrow

Tissue was collected, processed into a single cell suspension in sterile FACS buffer, and stained with antibodies against Ter119, CD71, c-KIT, CD45, DNA-binding dye Draq5, and RNA-binding dye thiozole orange to delineate the stages of RBC development. Images were collected with an Amnis Imagestream Mk II and analyzed with IDEAS software.

### Bone Marrow (BM) Chimeras

Four to 6-week-old B6.Thy1.1/1.2 and HOD.Thy1.1/Thy1.2 mice were given 2 doses of 600 cGY irradiation (RS 2000 Biological Irradiator, Rad Source Technologies) spaced 2-4 hours apart. Recipients were given 20 million bone marrow consisting of 10% of OTII.Rag2-/-.Thy1.2 + 10% of TCR75.Thy1.1 + 80% of bone marrow recipient genotype (either B6.Thy1.1/1.2 or HOD.Thy1.1/1.2) 24 hours post-irradiation, as described previously (21). HOD antibody production was analyzed by flow crossmatch.

### Statistical Analysis

Statistical significance was determined by one-way ANOVA with multiple comparisons post-test and p-value of ≤ 0.05 was considered significant; > 0.05 n.s., < 0.05 *, < 0.01 **, < 0.001 ***. Statistics were performed with GraphPad Prism.

## Results

### Murine Model of Primary Autoimmune Hemolytic Anemia (AIHA)

**Figure 1:**
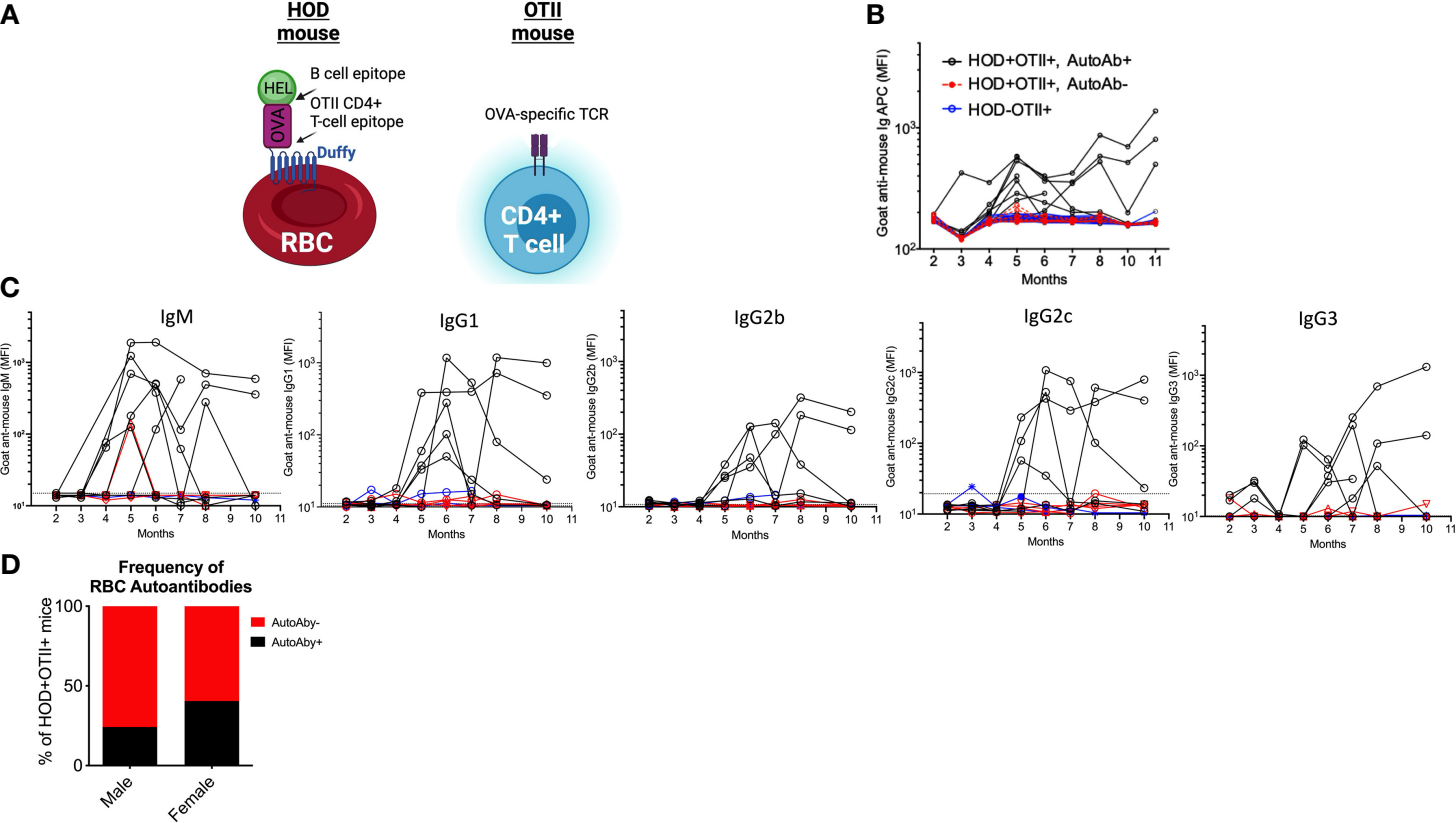
A subset of HOD+OTII+ mice develop RBC autoantibodies. **(A)** Graphical illustrations of the HOD RBC (left) and OTII T cells (right). Whole blood and sera were collected from HODxOTII F1 animals and analyzed monthly. HOD-specific autoantibodies were detected in sera by flow crossmatch and mean fluorescence intensity (MFI) of **(B)** total immunoglobulins (Igs) and **(C)** IgM and IgG subclasses were calculated. Two standard deviations from mean values of IgM and each IgG subtype of HOD-OTII+ mice were included in the graphs (dotted lines). **(D)** The frequency of HOD+OTII+ animals that made detectable RBC autoantibodies was determined and broken down by sex. Results are representative of 4 independent cohorts of HODxOTII F1 animals (n = 10 HOD-OTII+ and 10-20 HOD+OTII+ mice per cohort). Each line represents an individual mouse of a particular genotype and autoantibody status: HOD-OTII+ (blue circles), HOD+OTII+ without autoantibodies (red filled circles, dashed lines), and HOD+OTII+ with autoantibodies (black circles).

To study how red blood cell (RBC)-specific T cell tolerance is established and to identify which pathways are compromised when autoimmune disease develops, we generated a murine model of primary autoimmune hemolytic anemia (AIHA) by breeding HOD and OTII mice (HODxOTII F1 animals which included HOD+OTII+ autoimmune genotype and HOD-OTII+ non-autoimmune genotype littermate control animals) (**Figure 1A**). Sera was collected monthly from HODxOTII F1 animals and evaluated for production of detectable HOD RBC antibodies by flow crossmatch (**Figure 1B**). Analysis of serum from young HOD+OTII+ autoimmune genotype mice showed no RBC autoantibody production, which correlates with our previous data demonstrating that stringent tolerance is established to the HOD autoantigen (16). Unexpectedly, a subset of animals within the HOD+OTII+ autoimmune genotype cohort (red lines) developed age-related RBC autoantibodies (black lines), with an approximate onset of 4-5 months of age. Sera from many HODxOTII F1 animals were longitudinally analyzed out to 11 months of age. Detectable autoantibodies were both IgM and IgG, along with subclasses IgG1, IgG2b, IgG2c, and IgG3 (**Figure 1C**). Moreover, we observed 2 distinct groups of autoantibody-producing mice—relapse/remitting and sustained (**Figures 1B, C**). While none of the HOD-OTII+ littermate control animals (blue lines) made detectable anti-HOD antibodies, an average of 20% of male and 40% of female HOD+OTII+ mice made RBC autoantibodies (**Figure 1D**).

**Figure 2:**
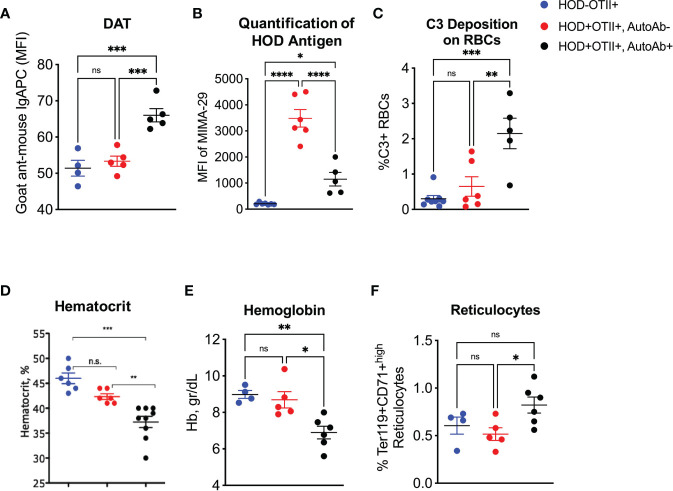
Effects of autoantibodies production on RBCs. Peripheral RBCs were collected and **(A)** evaluated for DAT, **(B)** HOD antigen expression and **(C)** C3 complement deposition. **(D)** Hematocrit, **(E)** hemoglobin and **(F)** reticulocytes were calculated from whole blood samples. Results are representative of 4 independent cohorts of HODxOTII F1 animals (n = 10 HOD-OTII+ and 10-20 HOD+OTII+ mice per cohort). HOD-OTII+ (blue circles), HOD+OTII+ without autoantibodies (red circles), and HOD+OTII+ with autoantibodies (black circles). Statistical significance was determined by one-way ANOVA with multiple comparisons post-test and p-value > 0.05 n.s., < 0.05 *, < 0.01 **, < 0.001 ***, < 0.0001 ****.

Binding of anti-erythrocyte autoantibodies can have a myriad of effects on RBCs, including antigen modulation, complement recruitment and hemolysis (13, 23–25). To test for autoantibody binding, we performed a direct anti-globulin test (DAT) with peripheral RBCs. RBCs from autoantibody-producing HOD+OTII+ animals had significantly higher levels of bound antibodies compared to controls (**Figure 2A**). In many instances, RBC-binding antibodies are non-hemolytic but can mediate antigen modulation. To test whether HOD antigen expression was reduced in autoantibody-producing HOD+OTII+ animals, peripheral RBCs were stained with MIMA-29, an antibody that recognizes Duffy within the HOD antigen (19). Detectable HOD antigen was significantly reduced on RBCs from HOD+OTII+ mice with autoantibodies, compared to HOD+OTII+ without autoantibodies (**Figure 2B**). No HOD expression was detected in HOD-OTII+ animals. In some instances, antibody recognition of erythrocyte antigens facilitates RBC destruction (i.e., hemolysis). To evaluate whether HOD-specific autoantibodies in HOD+OTII+ animals were pathogenic, C3 complement deposition, hematocrit, and hemoglobin was determined. RBCs from HOD+OTII+ mice with autoantibodies had elevated frequencies of RBCs with detectable C3 complement deposition (**Figure 2C**). Additionally, HOD+OTII+ mice with RBC autoantibodies had significantly lower hematocrit and hemoglobin values, compared to HOD-OTII+ littermate controls (**Figures 2D, E**). Autoantibody-producing HOD+OTII+ animals also had increased Ter119+CD71^high^ reticulocytes in peripheral blood (**Figure 2F**). The reduction in hematocrit and hemoglobin were likely in response to autoantibodies, and thereby autoantibody-mediated hemolysis, as HOD+OTII+ mice without autoantibodies had similar values as control animals. Together, these data indicate that RBC autoantibodies are affecting HOD RBCs by modulating antigen expression and facilitating hemolysis through complement binding, thereby resulting in reduced hematocrit and hemoglobin concentration. Further, these data suggest that RBC autoantibodies are leading to anemia and compensatory reticulocytosis.

**Figure 3:**
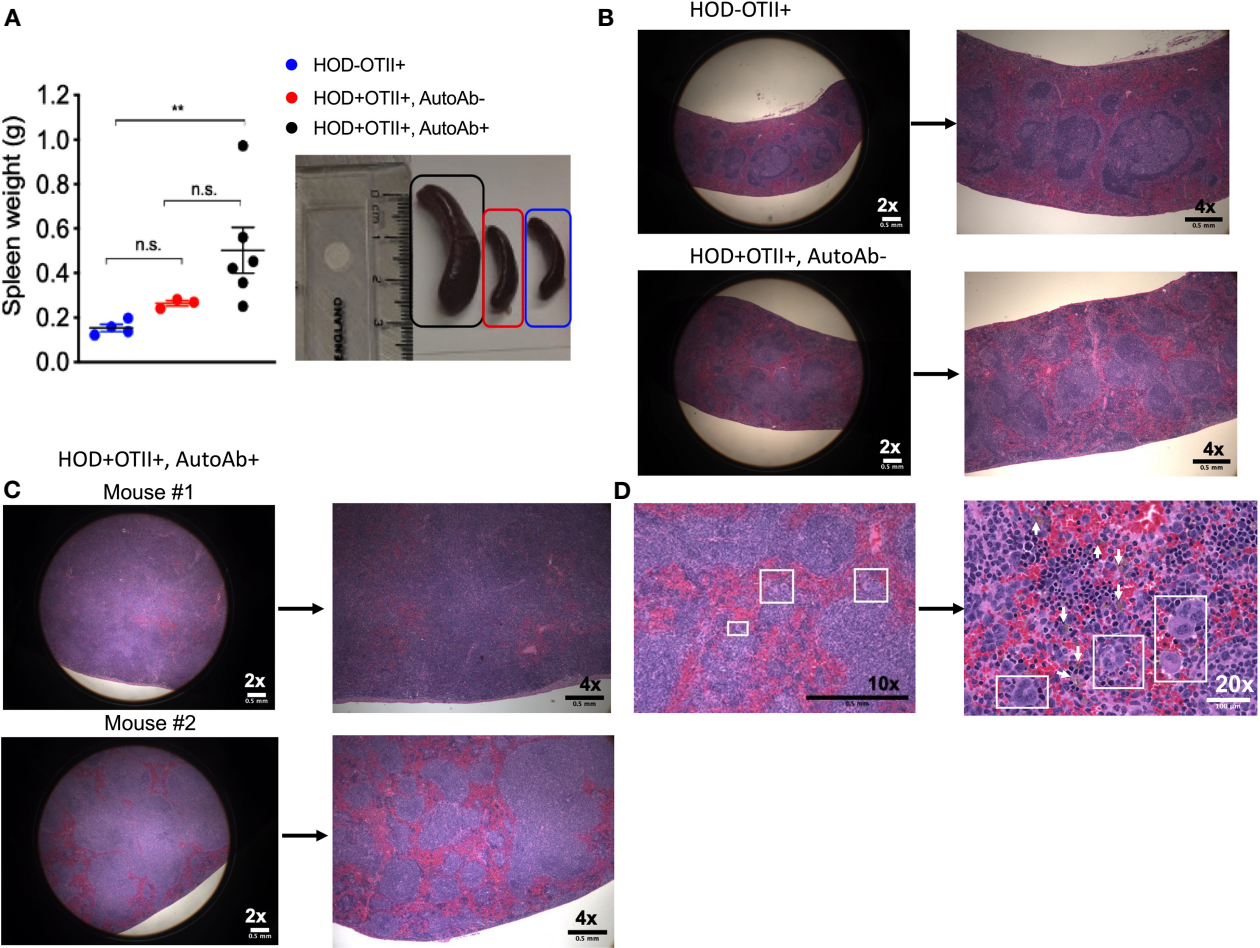
HOD+OTII+ mice have splenomegaly and disrupted splenic architecture. Spleens from aged HODxOTII F1 animals were collected and **(A)** weights were determined. To visualize splenic architecture, spleens were fixed, sectioned, and stained with H&E. **(B)** Spleens from HOD-OTII+ and HOD+OTII+ without autoantibodies and **(C)** HOD+OTII+ mice with autoantibodies are shown at 2x and 4x magnification. **(D)** In select splenic sections from HOD+OTII+ mice with autoantibodies, megakaryocyte-like cells were identified (boxes on 10x and 20x images) and arrows shown in the 20x image point to hemosiderin macrophages. Images are representative of 4 individual mice of each genotype and condition. Scale bars are indicated on each image. Statistical significance was determined by one-way ANOVA with multiple comparisons post-test and p-value > 0.05 n.s. and < 0.01 **.

**Figure 4:**
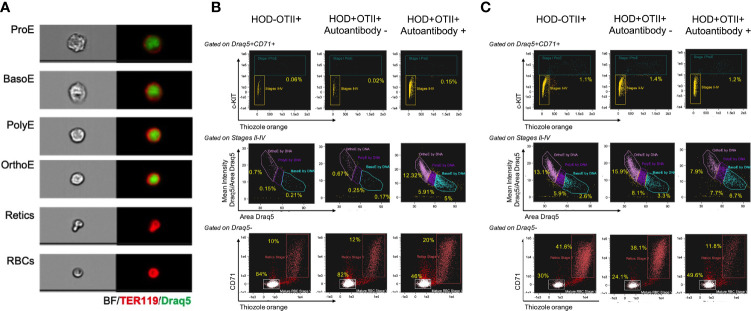
Autoantibody-producing HOD+OTII+ mice have extramedullary hematopoiesis. Spleens and bone marrow were collected from HODxOTII F1 animals, processed into a single cell suspension, and stained with antibodies to delineate RBC progenitors and development. **(A)** Representative images of each RBC developmental stage are shown. Flow plots and gating strategy used to identify each RBC progenitor in the **(B)** spleen and **(C)** bone marrow. Images are representative of 3 individual mice of each genotype and condition.

Patients with AIHA often have splenomegaly and compensatory extramedullary erythropoiesis. To test whether RBC autoantibodies in HOD+OTII+ mice elicited splenic erythropoiesis, age-matched animals were euthanized and spleens were collected for analysis. Spleens from HOD+OTII+ mice with RBC autoantibodies weighed significantly more and were larger than their non-autoantibody counterparts and littermate control animals (**Figure 3A**). Splenomegaly can be due to splenic accumulation/sequestration of RBCs and extramedullary hematopoiesis. As such, histologic analysis was performed. In H&E stained sections, both HOD-OTII+ littermate control mice and HOD+OTII+ animals without autoantibodies have a well-defined splenic arrangement, with clearly demarcated red and white pulp areas (**Figure 3B**). In contrast, HOD+OTII+ mice with autoantibodies had a severe distortion of normal splenic architecture, with enlarged white pulp areas and a correlating reduction in red pulp (**Figure 3C**). Additionally, large, megakaryocyte-like cells were observed, suggesting possible extramedullary erythropoiesis (**Figure 3D**, boxed areas). There were also notably more hemosiderin-containing macrophages present (**Figure 3D**, arrows). As H&E stain cannot readily delineate stages of RBC development, we utilized an antibody panel to discern RBC precursors to visualize with an Amnis Imagestream Mk II. As RBCs mature, the overall size reduces in conjunction with nuclear condensation (and correlating loss of residual DNA and RNA). Using size, nuclear content, and surface markers for RBCs, we developed an antibody panel and gating strategy to identify the stages of erythropoiesis: proerythroblasts (Pro-E), basophilic (Baso-E), polychromatophilic (Poly-E), orthochromatophilic (Ortho-E), reticulocytes, and mature RBCs (**Figure 4A** and **Supplementary Figure 1**) (26). Increased frequencies of all RBC progenitors (Ter119+Draq5+CD71+), but decreased frequencies of mature RBCs (Ter119+Draq5-CD71-), were evident in spleens collected from HOD+OTII+ mice with detectable RBC autoantibodies (**Figure 4B**). These data demonstrate that, upon RBC autoantibody production, extramedullary erythropoiesis occurred in the spleen. As extramedullary erythropoiesis is a compensatory mechanism for insufficient bone marrow-derived RBC production (27), evaluation of RBC precursors in the bone marrow revealed that production of autoantibodies correlated with reduced frequencies of progenitors (**Figure 4C**). Thus, in HOD+OTII+ animals with autoantibodies, erythropoiesis is dysregulated.

Although the underlying cause(s) of primary AIHA is unknown, failure in tolerance to RBC autoantigens has been associated with many factors, including infections and inflammation. To test whether altered cytokine production preceded autoantibody generation, plasma collected from animals prior to and after detection of RBC autoantibodies was assessed for proinflammatory cytokine levels (e.g., IL-6, IFN gamma, etc.). No significant differences were noted between HOD+OTII+ and autoantibody-producing HOD+OTII+ mice (**Supplementary Figure 2**). Moreover, there were no significant differences in cytokine levels between plasma collected before or after autoantibody production.

One limitation of HODxOTII F1 mice is the high frequency of CD4+ OTII T cells. To test if the observed phenotype was a consequence of the precursor frequency, we generated bone marrow chimeras in which B6 or HOD mice were irradiated and reconstituted with bone marrow consisting of 80% recipient genotype (B6.Thy1.1/1.2 or HOD.Thy1.1/1.2) + 10% OTII.Rag2-/-.Thy1.2 + 10% TCR75.Thy1.1 (a CD4+ TCR transgenic T cell with a specificity to an antigen not in this system) as previously described (21). Using this approach, a subset HOD.Thy1.1/1.2 autoreactive mice developed age-related onset autoimmunity (**Supplementary Figure 3**). Thus, decreased frequencies of autoreactive T cells did not prevent autoimmune disease. And, while we cannot rule out that autoreactive T cell frequency may facilitate tolerance failure, these data suggest that age-related changes may play a more prominent role.

## Discussion

Tolerance to autoantigens is required to prevent autoimmune disease. Failure of tolerance to RBCs can lead to AIHA, a severe and sometimes fatal disease. To gain insight into how tolerance to erythrocytes is established and broken, we utilized a well-described HOD RBC transgenic mouse (15). With this model, stringent T cell tolerance, but not B cell tolerance, was observed (16). By breeding HOD RBC-reactive OTII T cell transgenic mice with HOD animals, we developed a model of RBC autoreactivity whereby young HOD+OTII+ mice displayed stringent T cell tolerance without detectable autoantibodies (21); however, upon aging, a subset of mice developed age-related erythrocyte-specific autoantibodies. Similar to observations in humans with AIHA, our mouse model reflects primary AIHA including increased incidence with age, female predominance, hemolysis and anemia, and extramedullary erythropoiesis.

In both observations in humans and murine models, RBC-binding antibodies have been shown to mediate a wide-range of effects on RBCs, including antigen modulation and hemolysis (9, 13, 28–32). Autoantibodies detected in HOD+OTII+ mice were pathogenic, leading to both RBC antigen loss and evidence of hemolysis, as determined by significant reductions in hematocrit and hemoglobin. Additionally, there were 2 subsets of animals within the autoantibody-producing cohort—those that had relapse/remitting autoantibodies and those that had sustained levels. This is also a common feature of pathology observed in humans with AIHA (33). Importantly, antibody isotype and subtype may dictate the severity of AIHA disease; human IgG1 is the most frequently detected subclass in humans with AIHA and this autoantibody subtype may be found together with C3 complement deposition on the patient’s RBCs (9, 33). Likewise, multiple murine IgG subclasses can fix complement (e.g., IgG3, IgG2b, and IgG2a/c) (34), and both autoantibodies (of multiple subclasses) and C3 were detected in HOD+OTII+ animals. Importantly, our group has previously shown that HOD-specific IgG2c mediates rapid clearance of HOD RBCs, leads to robust alloantibodies upon transfusion, and promotes memory lymphocyte formation (35). Thus, the presence of this potent pro-inflammatory autoantibody subclass could perpetuate RBC hemolysis, thereby exacerbating AIHA symptoms. However, autoantibody persistence could not be associated with the presence/absence of a particular antibody isotype or subclass. Moreover, although HOD+OTII+ animals with autoantibodies had significantly higher DAT MFIs compared to controls, these values were very low, despite measurement at multiple timepoints throughout aging. Whilst DAT negative AIHA cases occur in 5-10% of humans with AIHA, these cases are considered atypical. It is plausible, however, that the kinetics of autoantibody binding to the HOD antigen followed by the removal of autoantigen would prevent it from being detected with our assays. Alternatively, of the RBCs that retain HOD expression, antigen may be rapidly removed due to autoantibody binding and recruitment of C3. Finally, given the low levels of remaining HOD antigen detectable on RBC in mice with autoantibodies, it is plausible that there are no appreciable amounts of autoantigen to bind; thus, high levels of autoantibodies remain in the sera.

Similar to human patients with AIHA, mice developed anemia (shown as a decreased hematocrit percentage, reduced hemoglobin concentration and increased reticulocytes) (**Figures 2**, **4**). However, how and if the development of anemia is a consequence of the influence of autoantibody kinetics still remain open questions. Anemia and cytopenia can cause splenomegaly and splenic extramedullary hematopoiesis, both of which are common clinical presentations in patients with AIHA (36–38). Splenomegaly and extramedullary erythropoiesis are both observed in HOD+OTII+ mice with autoantibodies, compared to controls (**Figure 3**, **4**). The spleen is the site of clearance of senescent RBCs and is also a secondary lymphoid organ capable of promoting adaptive immune responses. In the presence of RBC-specific antibodies, the spleen can become congested due to accumulation of RBCs. Indeed, Chadebech et al. (39) published splenic histopathology in an IgA-mediated AIHA case and showed marked RBC sequestration with atrophic white pulp but low levels of erythrophagocytosis. Similar findings have been observed in the NZB murine model of AIHA (40). Notably, the splenic architecture was severely distorted in HOD+OTII+ mice with autoantibodies. Whilst normal-sized germinal centers were observed in control animals, mice with autoantibodies had lymphocytosis, with nearly diminished red pulp areas. This observation could be due to the age of the mice (spleens are reflective of 8-10 months of age) in combination with the persistence of autoantibody levels, or simply an aggressive B cell response correlating with high autoantibody titers. Other noteworthy observations were the presence of RBC progenitors and megakaryocyte-like cells, which are both indicative of extramedullary erythropoiesis. Extramedullary erythropoiesis may be in the response to the ongoing hemolysis and/or the insufficient production of RBCs in the bone marrow (**Figure 4C**).

Whilst several factors have been hypothesized to initiate idiopathic AIHA (e.g., genetics, environment, etc.), the underlying cause(s) is unknown. There was not an observable association between tolerance failure and infection/pathogen exposure or microbiome. In particular, this model was initially established at BloodworksNW (Seattle, WA) and then re-established at Columbia University (New York City, NY), with similar trends in frequencies of autoantibody production and female predominance. While both vivarium are specific pathogen free, there were marked differences in the pathogen composition and potential exposure. Additionally, no significant changes in inflammatory status was observed as cytokine analysis showed no differences between HOD+OTII+ autoimmune genotype mice regardless of autoantibody status (**Supplementary Figure 2**). Moreover, not all animals from the same litter or those co-housed together developed autoantibodies. Given the importance of the mother’s microbiome in offspring gut flora and that mice are coprophagic, these observations suggests that a microbiome-independent pathway may be responsible for the failure of T cell tolerance. As a consistent thread in observations in humans with autoimmune disease and murine models of autoimmunity, there is an age-related component to development of AIHA in HOD+OTII+ animals. Both immunosenescence and inflammaging are postulated to contribute to immune dysregulation and autoimmunity upon aging (41–43). As such, because our model allows for easy identification of autoreactive T cells, it will be useful in interrogating T cell development and function as mice age and some develop autoantibodies.

As T cell tolerance is the stopgap to RBC autoimmunity in this model, one limitation of the studies presented is the supraphysiological frequency of autoreactive T cells. To address this, we generated bone marrow chimeras whereby the precursor frequency of autoreactive CD4+ T cells was significantly reduced. Similar to observations with the HODxOTII F1 animals, young bone marrow chimera mice displayed robust T cell tolerance, with multiple peripheral tolerance mechanisms employed (21). However, also similar to HODxOTII F1 animals, as bone marrow chimera animals aged, a subset of animals produced anti-erythrocyte autoantibodies (**Supplementary Figure 3**). Thus, even at significantly reduced frequencies of autoreactive T cells, T cell tolerance fails in a subset of animals. As irradiation itself can promote autoimmunity (44), all additional studies have utilized the HODxOTII F1 animals as the preferable murine model of idiopathic AIHA.

**Figure 5:**
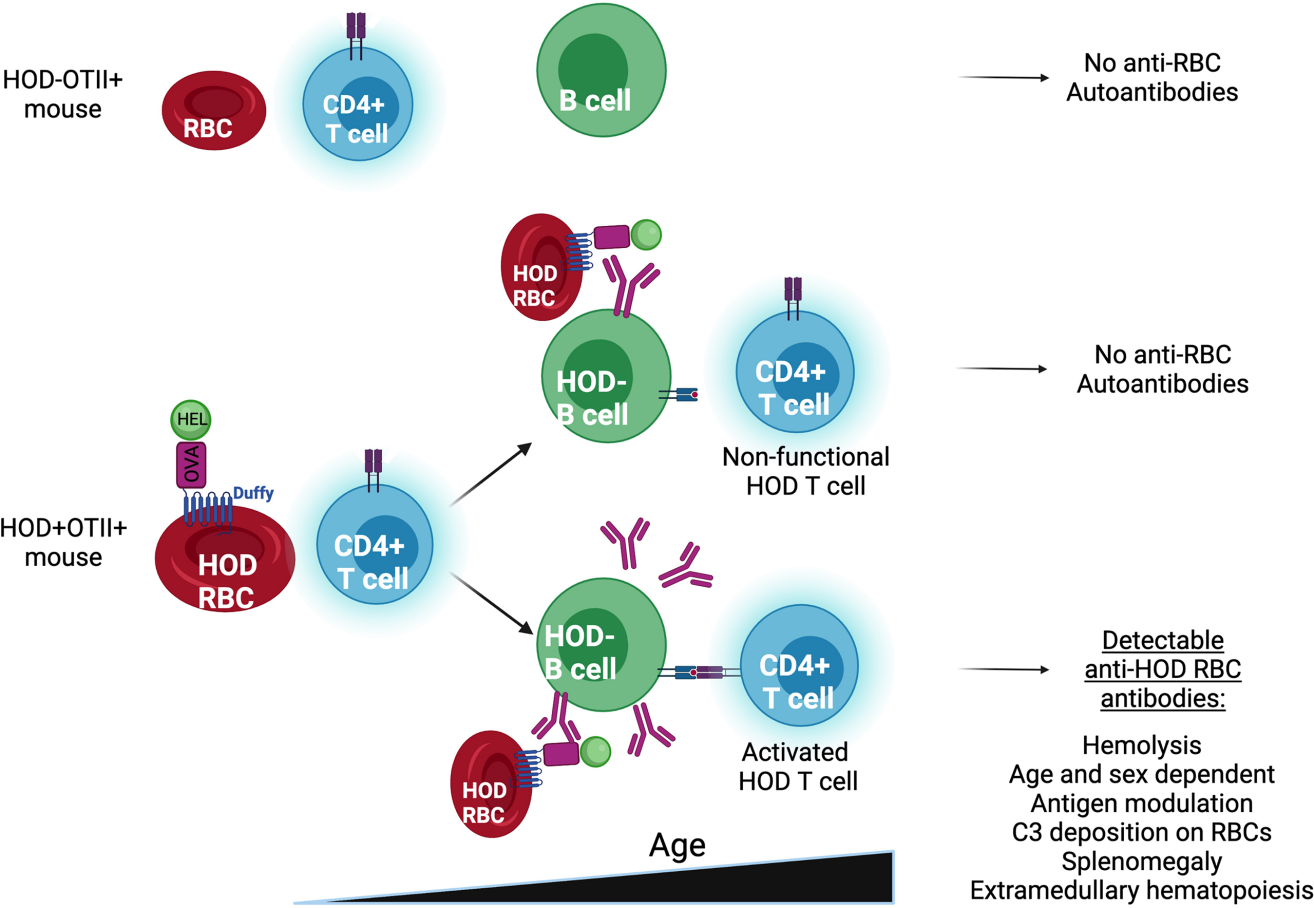
Representing scheme of HOD-OTII, HOD+OTII+ without and with Autoantibodies.

In summary, herein we describe a new murine model of AIHA which shares many characteristics with primary AIHA in humans, including female predominance, age-related onset, hemolysis and anemia, splenomegaly, and extramedullary erythropoiesis (**Figure 5**). Because of the antigens used in the development of this model, there are numerous immunological tools available to dissect which T cell tolerance pathways are required to prevent autoimmunity. As such, this model can be used to provide new insights into the underlying mechanisms in the initiation of idiopathic AIHA.

## Data Availability Statement

The original contributions presented in the study are included in the article/**Supplementary Material**. Further inquiries can be directed to the corresponding author.

## Ethics Statement

The animal study was reviewed and approved by BloodworksNW IACUC and Columbia University IACUC.

## Author Contributions

KH and FD designed the studies and experiments. FD, AQ, FL, CM, and KH carried out experiments. All authors were involved in interpretation of data. FD and KH wrote the manuscript. All authors contributed to the article and approved the submitted version.

## Funding

This work was supported, in part, by grants to KH from the National Institutes of Health (NHLBI R01HL133325) and the National Blood Foundation.

## Conflict of Interest

The authors declare that the research was conducted in the absence of any commercial or financial relationships that could be construed as a potential conflict of interest.

## Publisher’s Note

All claims expressed in this article are solely those of the authors and do not necessarily represent those of their affiliated organizations, or those of the publisher, the editors and the reviewers. Any product that may be evaluated in this article, or claim that may be made by its manufacturer, is not guaranteed or endorsed by the publisher.

## Supplementary Material

Supplementary Figure 1 Amnis ImageStream Mk II gating strategy. Gating strategy for identification of RBC progenitors in murine splenocytes using the Amnis Imagestreatm.

Supplementary Figure 2 Cytokine quantification in HODxOTII F1 animals. Sera was collected longitudinally from HODxOTII F1 animals. Pro- and anti-inflammatory cytokines were assessed before and after autoantibody production. HOD+OTII+ mice with (black = 7) and without (red, n = 6) autoantibodies are shown. Data are presented as mean +/- SEM.

Supplementary Figure 3 HOD mice with reduced autoreactive T cell frequencies make autoantibodies. B6.Thy1.1/1.2 and HOD.Thy1.1/1.2 recipients were irradiated and reconstituted with a mix of bone marrow: 10% OTII.Rag2^-/-^.Thy1.2, 10% TCR75.Thy1.1, and either 80% B6.Thy1.1/1.2 or 80% HOD.Thy1.1/1.2, as described in (21). Upon reconstitution, bone marrow chimeric mice were monitored longitudinally for autoantibody production by flow crossmatch. Flow crossmatch data presented are shown as the fold change in experimental serum recognizing and staining HOD targets compared to control B6 targets.

## References

[1] Gehrs BC Friedberg RC. Autoimmune Hemolytic Anemia. Am J Hematol (2002) 69(4):258–71. doi: 10.1002/ajh.10062 11921020

[2] Brodsky RA. Warm Autoimmune Hemolytic Anemia. New Engl J Med (2019) 381(7):647–54. doi: 10.1056/NEJMcp1900554 31412178

[3] Quintero OL Amador-Patarroyo MJ Montoya-Ortiz G Rojas-Villarraga A Anaya J-M. Autoimmune Disease and Gender: Plausible Mechanisms for the Female Predominance of Autoimmunity. J Autoimmun (2012) 38(2):J109–19. doi: 10.1016/j.jaut.2011.10.003 22079680

[4] Go RS Winters JL Kay NE. How I Treat Autoimmune Hemolytic Anemia. Blood (2017) 129(22):2971. doi: 10.1182/blood-2016-11-693689 28360039

[5] Lechner K Jäger U. How I Treat Autoimmune Hemolytic Anemias in Adults. Blood (2010) 116(11):1831. doi: 10.1182/blood-2010-03-259325 20548093

[6] Hill A Hill QA. Autoimmune Hemolytic Anemia. Hematol Am Soc Hematol Educ Program (2018) 2018(1):382–9. doi: 10.1182/asheducation-2018.1.382 PMC6246027 30504336

[7] Vos GH Petz LD Hugh Fudenberg H. Specificity and Immunoglobulin Characteristics of Autoantibodies in Acquired Hemolytic Anemia. J Immunol (1971) 106(5):1172. 4995749

[8] Chaudhary RK Das SS. Autoimmune Hemolytic Anemia: From Lab to Bedside. Asian J Transfusion Sci (2014) 8(1):5–12. doi: 10.4103/0973-6247.126681 PMC3943148 24678166

[9] Barcellini W Zaninoni A Giannotta JA Fattizzo B. New Insights in Autoimmune Hemolytic Anemia: From Pathogenesis to Therapy. J Clin Med (2020) 9(12):3859. doi: 10.3390/jcm9123859 PMC7759854 33261023

[10] Rao VK Oliveira JB. How I Treat Autoimmune Lymphoproliferative Syndrome. Blood (2011) 118(22):5741–51. doi: 10.1182/blood-2011-07-325217 PMC3228494 21885601

[11] Garratty G. The Significance of IgG on the Red Cell Surface. Transfusion Med Rev (1987) 1(1):47–57. doi: 10.1016/S0887-7963(87)70005-4 2980266

[12] Zantek ND Koepsell SA Tharp DR Jr. Cohn CS. The Direct Antiglobulin Test: A Critical Step in the Evaluation of Hemolysis. Am J Hematol (2012) 87(7):707–9. doi: 10.1002/ajh.23218 22566278

[13] />Howie HL Hudson KE. Murine Models of Autoimmune Hemolytic Anemia. Curr Opin Hematol (2018) 25(6):473–81. doi: 10.1097/MOH.0000000000000459 PMC6200381 30169458

[14] Vickers MA Barker RN. Chapter 47 - Autoimmune Hemolytic Anemia. In: Rose NR Mackay IR, editors. The Autoimmune Diseases, Sixth Edition. Aberdeen, UK: Academic Press (2020). p. 897–910.

[15] Desmarets M Cadwell CM Peterson KR Neades R Zimring JC. Minor Histocompatibility Antigens on Transfused Leukoreduced Units of Red Blood Cells Induce Bone Marrow Transplant Rejection in a Mouse Model. Blood (2009) 114(11):2315–22. doi: 10.1182/blood-2009-04-214387 PMC2745850 19525479

[16] Hudson KE Hendrickson JE Cadwell CM Iwakoshi NN Zimring JC. Partial Tolerance of Autoreactive B and T Cells to Erythrocyte-Specific Self-Antigens in Mice. Haematologica (2012) 97(12):1836–44. doi: 10.3324/haematol.2012.065144 PMC3590090 22733018

[17] Glatigny S Bettelli E. Experimental Autoimmune Encephalomyelitis (EAE) as Animal Models of Multiple Sclerosis (Ms). Cold Spring Harbor Perspect Med (2018) 8(11). doi: 10.1101/cshperspect.a028977 PMC6211376 29311122

[18] Fontes JA Barin JG Talor MV Stickel N Schaub J Rose NR. Complete Freund’s Adjuvant Induces Experimental Autoimmune Myocarditis by Enhancing IL-6 Production During Initiation of the Immune Response. Immunity Inflammation Dis (2017) 5(2):163–76. doi: 10.1002/iid3.155 PMC5418134 28474508

[19] Richards AL Kapp LM Wang X Howie HL Hudson KE. Regulatory T Cells Are Dispensable for Tolerance to RBC Antigens. Front Immunol (2016) 7:348. doi: 10.3389/fimmu.2016.00348 27698653 PMC5027202

[20] Barnden MJ Allison J Heath WR Carbone FR. Defective TCR Expression in Transgenic Mice Constructed Using cDNA-Based α- and β-Chain Genes Under the Control of Heterologous Regulatory Elements. Immunol Cell Biol (1998) 76(1):34–40. doi: 10.1046/j.1440-1711.1998.00709.x 9553774

[21] Wong ASL Gruber DR Richards AL Sheldon K Qiu A Hay A. Tolerization of Recent Thymic Emigrants is Required to Prevent RBC-Specific Autoimmunity. J Autoimmun (2020) 114:102489. doi: 10.1016/j.jaut.2020.102489 32507505 PMC7572580

[22] Honjo K Xu Xy Bucy RP. CD4+ T-Cell Receptor Transgenic T Cells Alone can Reject Vascularized Heart Transplants Through the Indirect Pathway of Alloantigen Recognition. Transplantation (2004) 77(3):452–5. doi: 10.1097/01.TP.0000112937.12491.42 14966425

[23] Zimring JC Cadwell CM Spitalnik SL. Antigen Loss From Antibody-Coated Red Blood Cells. Transfusion Med Rev (2009) 23(3):189–204. doi: 10.1016/j.tmrv.2009.03.002 19539874

[24] Richards AL Howie HL Kapp LM Hendrickson JE Zimring JC Hudson KE. Innate B-1 B Cells Are Not Enriched in Red Blood Cell Autoimmune Mice: Importance of B Cell Receptor Transgenic Selection. Front Immunol (2017) 8:1366. doi: 10.3389/fimmu.2017.01366 29163471 PMC5675845

[25] Berentsen S. Role of Complement in Autoimmune Hemolytic Anemia. Transfus Med Hemother (2015) 42(5):303–10. doi: 10.1159/000438964 PMC4678321 26696798

[26] Moras M Lefevre SD Ostuni MA. From Erythroblasts to Mature Red Blood Cells: Organelle Clearance in Mammals. Front Physiol (2017) 8:1076. doi: 10.3389/fphys.2017.01076 29311991 PMC5742207

[27] Sohawon D Lau Kk Lau T Bowden DK. Extra-Medullary Haematopoiesis: A Pictorial Review of its Typical and Atypical Locations. (1754-9485 (Electronic)). eng. J Med Imaging Radiat Oncol (2012) 56(5):538–44. doi: 10.1111/j.1754-9485.2012.02397.x 23043573

[28] Seyfried H Górska B Maj S Sylwestrowicz T Giles CM Goldsmith KLG. Apparent Depression of Antigens of the Kell Blood Group System Associated With Autoimmune Acquired Haemolytic Anaemia. Vox Sanguinis (1972) 23(6):528–36. doi: 10.1111/j.1423-0410.1972.tb03846.x 4657687

[29] Marsh Wl Oyen R Alicea E Linter M Horton S. Autoimmune Hemolytic Anemia and the Kell Blood Groups. (0361-8609 (Print)). Am J Hematol (1979) 7(2):155–62. doi: 10.1002/ajh.2830070208 539592

[30] Vengelen-Tyler V Gonzalez B Garratty G Kruppe C Johnson CL Mueller KA. Acquired Loss of Red Cell Kell Antigens. Br J Haematol (1987) 65(2):231–4. doi: 10.1111/j.1365-2141.1987.00199.x-i1 3828231

[31] Zimring JC Hair GA Chadwick TE Deshpande SS Anderson KM Hillyer CD. Nonhemolytic Antibody-Induced Loss of Erythrocyte Surface Antigen. Blood (2005) 106(3):1105–12. doi: 10.1182/blood-2005-03-1040 15831698

[32] Zimring JC Cadwell CM Chadwick TE Spitalnik SL Schirmer DA Wu T. Nonhemolytic Antigen Loss From Red Blood Cells Requires Cooperative Binding of Multiple Antibodies Recognizing Different Epitopes. Blood (2007) 110(6):2201–8. doi: 10.1182/blood-2007-04-083097 17569819

[33] Barcellini W. New Insights in the Pathogenesis of Autoimmune Hemolytic Anemia. Transfusion Med Hemother (2015) 42(5):287–93. doi: 10.1159/000439002 PMC4678320 26696796

[34] Collins AM. IgG Subclass Co-Expression Brings Harmony to the Quartet Model of Murine IgG Function. (1440-1711 (Electronic)). eng. Immunol Cell Biol (2016) 94(10):949–54. doi: 10.1038/icb.2016.65 27502143

[35] Gruber DR Richards AL Howie HL Hay AM Lebedev JN Wang X. Passively Transferred IgG Enhances Humoral Immunity to a Red Blood Cell Alloantigen in Mice. Blood Adv (2020) 4(7):1526–37. doi: 10.1182/bloodadvances.2019001299 PMC7160277 32289162

[36] Farolino Dl Rustagi PK Currie MS Doeblin TD Logue GL. Teardrop-Shaped Red Cells in Autoimmune Hemolytic Anemia. (0361-8609 (Print)). eng. Am J Hematol (1986) 21(4):415–8. doi: 10.1002/ajh.2830210410 3953560

[37] Anguiano-Álvarez VM Hernández-Company A Hamdan-Pérez N Montante-M D Zúñiga-Tamayo DA Rodríguez-Rodríguez S. Splenic Myeloid Metaplasia in Warm Autoimmune Hemolytic Anemia (wAIHA): A Retrospective Study. Blood Res (2018) 53(1):35–40. doi: 10.5045/br.2018.53.1.35 29662860 PMC5898992

[38] Alwar V Shanthala DAM Sitalakshmi S Karuna RK. Clinical Patterns and Hematological Spectrum in Autoimmune Hemolytic Anemia. J Lab Phys (2010) 2(1):17–20. doi: 10.4103/0974-2727.66703 PMC3147080 21814401

[39] Chadebech P Michel M Janvier D Yamada K Copie-Bergman C Bodivit G. IgA-Mediated Human Autoimmune Hemolytic Anemia as a Result of Hemagglutination in the Spleen, But Independent of Complement Activation and Fcαri. Blood (2010) 116(20):4141–7. doi: 10.1182/blood-2010-03-276162 20644119

[40] Scatizzi JC Haraldsson MK Pollard KM Theofilopoulos AN Kono DH. The Lbw2 Locus Promotes Autoimmune Hemolytic Anemia. J Immunol (2012) 188(7):3307. doi: 10.4049/jimmunol.1103561 22371393 PMC3311724

[41] Thomas R Wang W Su D-M. Contributions of Age-Related Thymic Involution to Immunosenescence and Inflammaging. Immun Ageing (2020) 17(1):2. doi: 10.1186/s12979-020-0173-8 31988649 PMC6971920

[42] Goronzy JJ Weyand CM. Immune Aging and Autoimmunity. Cell Mol Life Sci (2012) 69(10):1615–23. doi: 10.1007/s00018-012-0970-0 PMC4277694 22466672

[43] Fukushima Y Minato N Hattori M. The Impact of Senescence-Associated T Cells on Immunosenescence and Age-Related Disorders. Inflammation Regeneration (2018) 38(1):24. doi: 10.1186/s41232-018-0082-9 30603051 PMC6304761

[44] Wittenborn TR Fahlquist Hagert C Ferapontov A Fonager S Jensen L Winther G. Comparison of Gamma and X-Ray Irradiation for Myeloablation and Establishment of Normal and Autoimmune Syngeneic Bone Marrow Chimeras. PloS One (2021) 16(3):e0247501. doi: 10.1371/journal.pone.0247501 33730087 PMC7968675

