# Supplementary figures and images for: A New Murine Model of Primary Autoimmune Hemolytic Anemia (AIHA)
Source: Front Immunol. 2021 Nov 15;12:752330. doi: 10.3389/fimmu.2021.752330 (PMC8634489; doi:10.3389/fimmu.2021.752330)

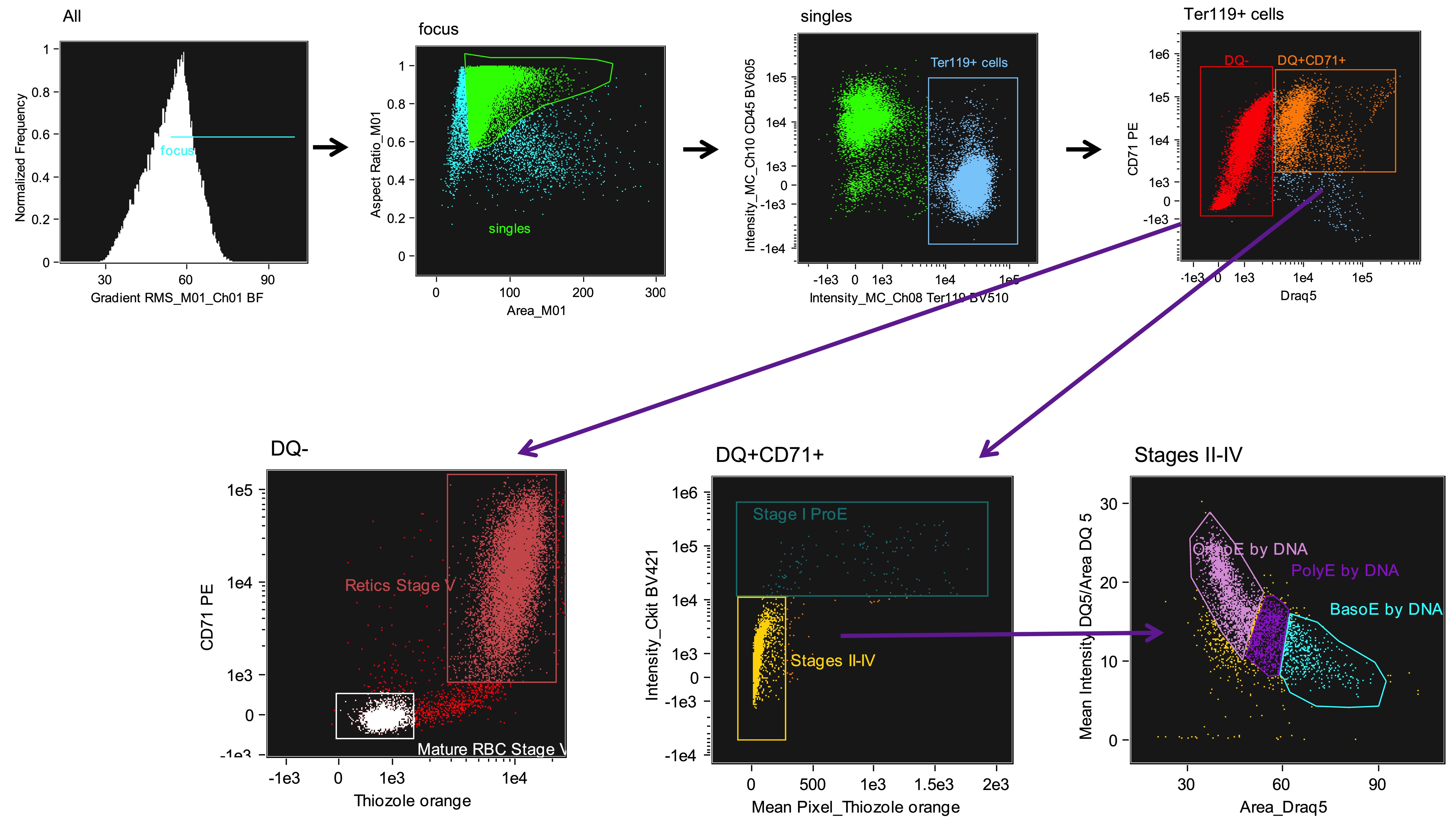

Supplement: Supplementary Figure 1 — Amnis ImageStream Mk II gating strategy. Gating strategy for identification of RBC progenitors in murine splenocytes using the Amnis Imagestreatm. [file Image_1.jpg]

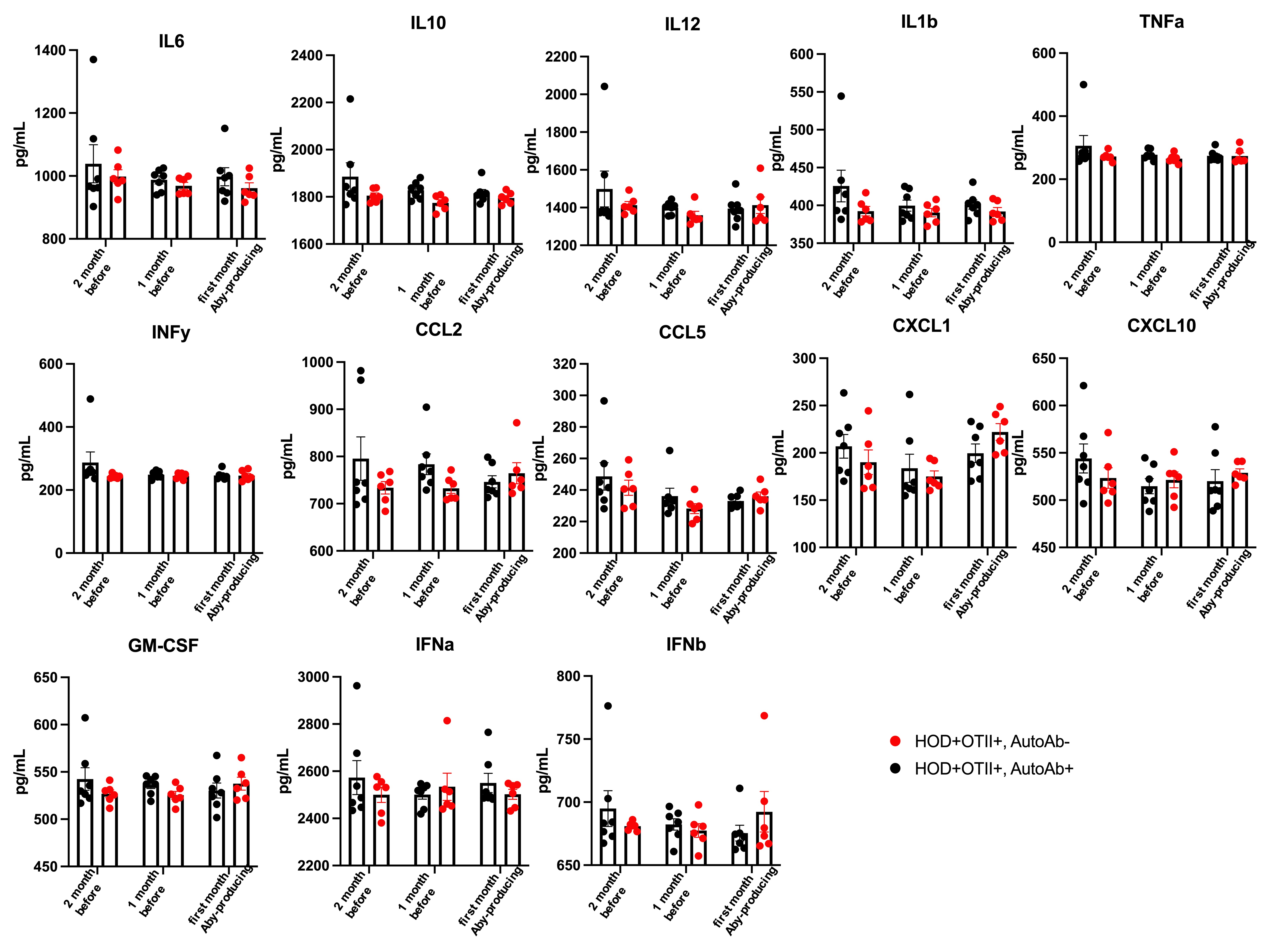

Supplement: Supplementary Figure 2 — Cytokine quantification in HODxOTII F1 animals. Sera was collected longitudinally from HODxOTII F1 animals. Pro- and anti-inflammatory cytokines were assessed before and after autoantibody production. HOD+OTII+ mice with (black = 7) and without (red, n = 6) autoantibodies are shown. Data are presented as mean +/- SEM. [file Image_2.jpg]

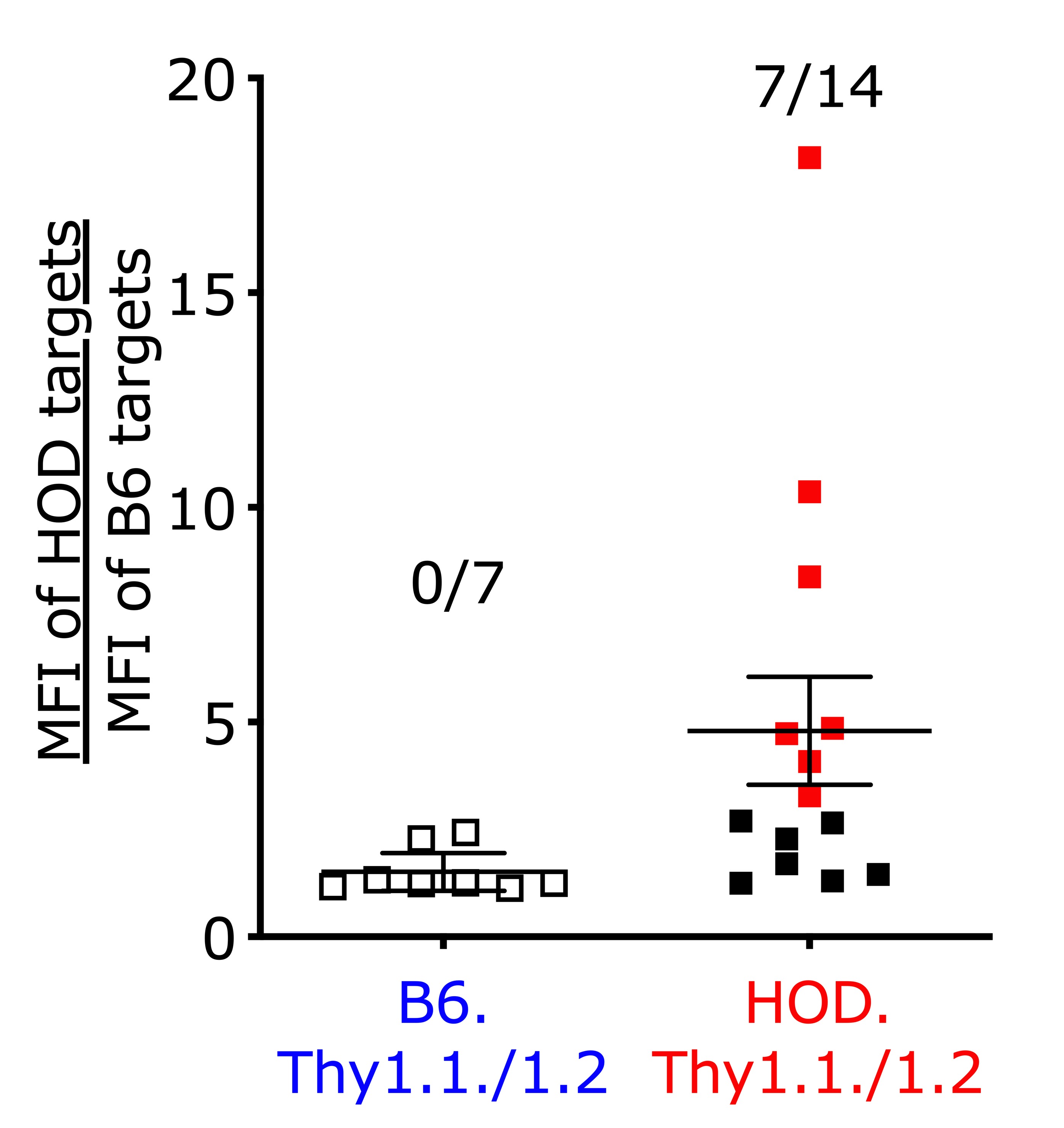

Supplement: Supplementary Figure 3 — HOD mice with reduced autoreactive T cell frequencies make autoantibodies. B6.Thy1.1/1.2 and HOD.Thy1.1/1.2 recipients were irradiated and reconstituted with a mix of bone marrow: 10% OTII.Rag2-/-.Thy1.2, 10% TCR75.Thy1.1, and either 80% B6.Thy1.1/1.2 or 80% HOD.Thy1.1/1.2, as described in (21). Upon reconstitution, bone marrow chimeric mice were monitored longitudinally for autoantibody production by flow crossmatch. Flow crossmatch data presented are shown as the fold change in experimental serum recognizing and staining HOD targets compared to control B6 targets. [file Image_3.jpg]
